# The Challenges of Approaching a Giant Breast Mass: A Case Report With Literature Review

**DOI:** 10.7759/cureus.77839

**Published:** 2025-01-22

**Authors:** Sayed Meshal A Ebrahim, Ahmed A Almass, Abdulhamid A Alarbash, Fozan A Aldulaijan

**Affiliations:** 1 General Surgery, Cairo University, Cairo, EGY; 2 General Surgery, King Fahd Hospital of the University, Imam Abdulrahman Bin Faisal University, Khobar, SAU; 3 Breast and Endocrine Surgery, King Fahad Specialist Hospital, Dammam, SAU

**Keywords:** breast sarcomatoid carcinoma, giant breast mass, leiomyosarcoma of the breast, malignant phyllodes tumor, triple assessment

## Abstract

The classical clinical approach to any breast lesion is through the triple assessment: clinical, radiological, and pathological. However, with giant breast masses, the classical assessment might have limitations in revealing the diagnosis. We report a case of a rapidly growing left breast mass in a 60-year-old woman. In a short period of time, the lesion reached a giant size of 48 cm x 43 cm with mixed constructure and skin changes. The lesion was considered malignant by triple assessment, but the core needle biopsy could not reach a single pathological diagnosis.

Giant breast masses are a rare entity of breast lesions that include different benign and malignant diseases. These diseases include malignant forms of breast sarcomas, phyllodes, and leiomyosarcomas, which are aggressive diseases requiring combative management. Diagnosing a giant breast tumor is challenging and demands a high level of suspicion. The classical approach may fail, raising the need for a special approach for such lesions. The adjusted guidelines may involve early multidisciplinary team involvement, advanced imaging, precise biopsy techniques, and earlier neoadjuvant therapy planning.

## Introduction

Triple assessment that includes clinical, radiological, and pathological assessment has shown great capability in diagnosing and directing the management of any breast lesion [1]. However, in some cases, especially with giant masses that have different types of differentiation, even triple assessment might face challenges in reaching a diagnosis [2-4]. Giant breast tumors are fast-growing breast masses with a weight of 500 gm or more and/or exceeding 5 cm in diameter. They can sometimes reach an enormous size, which causes skin congestion and ulceration due to centrifugal pressure [5]. Here, we report a case of a rapidly growing left breast mass in a 60-year-old woman with a discussion about similar challenging cases. This report is documented in line with the surgical case report (SCARE) 2023 guidelines [6].

## Case presentation

A 60-year-old Saudi single, postmenopausal woman with no known medical illnesses presented with a left breast mass progressively growing for the last five months with no skin or nipple changes. She had no complaints of weight loss, night sweats, or fever. Her family history is positive for breast cancer in her sister. Her past surgical history includes abdominal mass excision 30 years ago.

**Figure 1:**
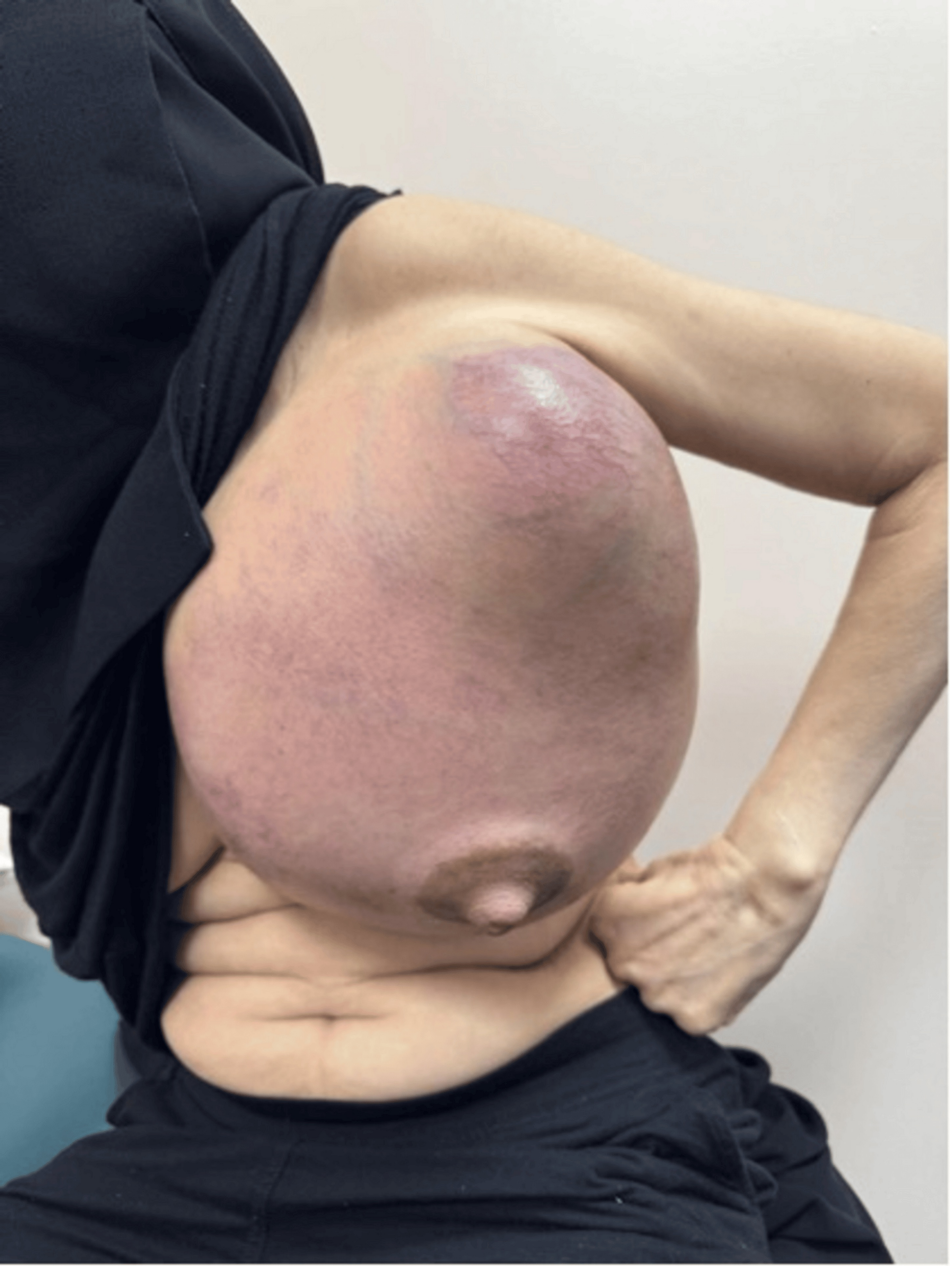
The huge left breast mass viewed from the anterior aspect.

**Figure 2:**
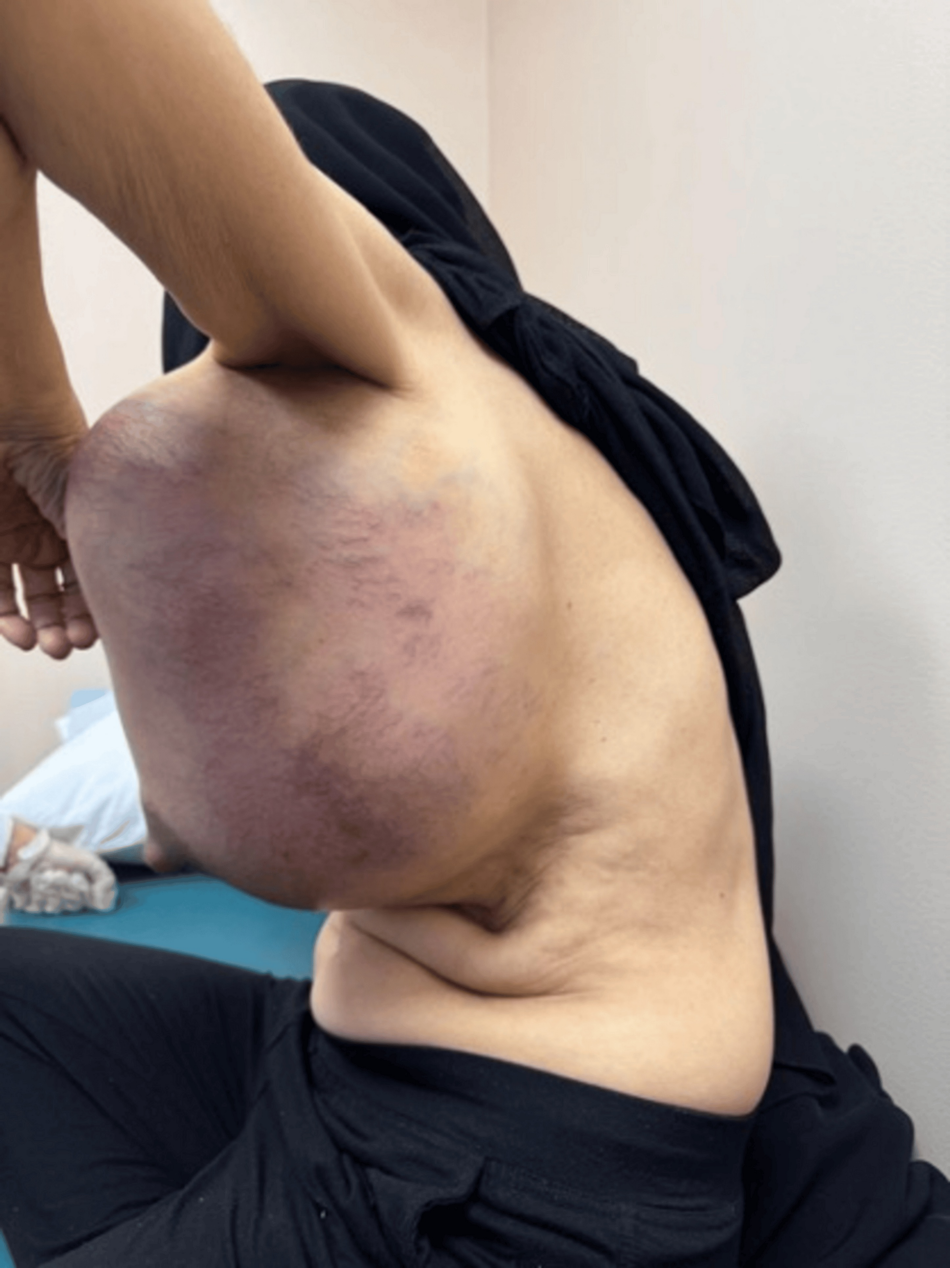
The huge left breast mass viewed from the lateral aspect.

The left breast examination showed a huge mass occupying the whole breast. It was around 48 cm x 43 cm, extending from the midline over the sternum to beyond the left posterior axillary line. It was a mix of soft and hard components, hot on palpation without tenderness. The overlying skin showed redness and bruises with prominent blood vessels (Figures 1-2). Her right breast examination showed a breast size A with grade 2 nipple ptosis (breast size was measured according to the cup size, while the ptosis was per Regnault’s classification [7,8]) with a small benign-looking mass. Both axillae were unremarkable.

**Figure 3:**
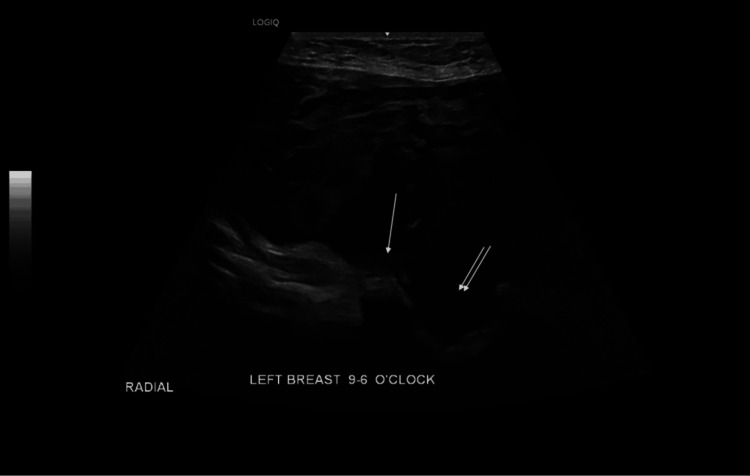
Left breast ultrasound showing mixed cystic and solid lesions The single arrow points at the solid component of the lesion, and the double arrows point at the cystic component of the lesion.

**Figure 4:**
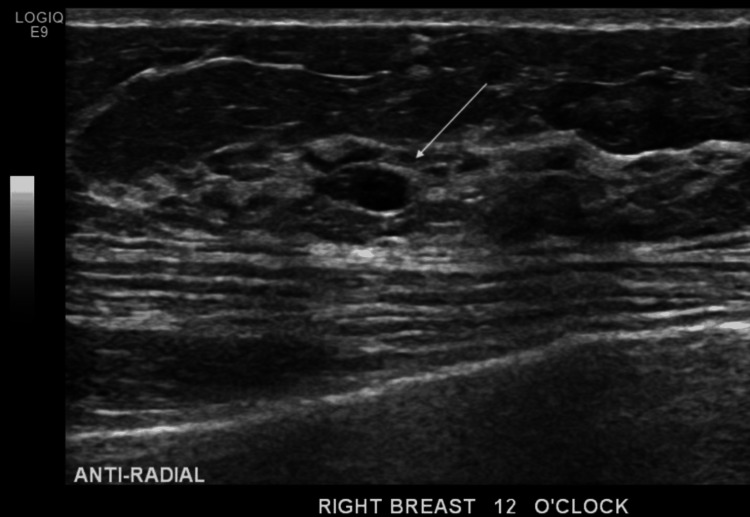
Ultrasound of the right breast showing a fibroadenoma (arrow)

**Figure 5:**
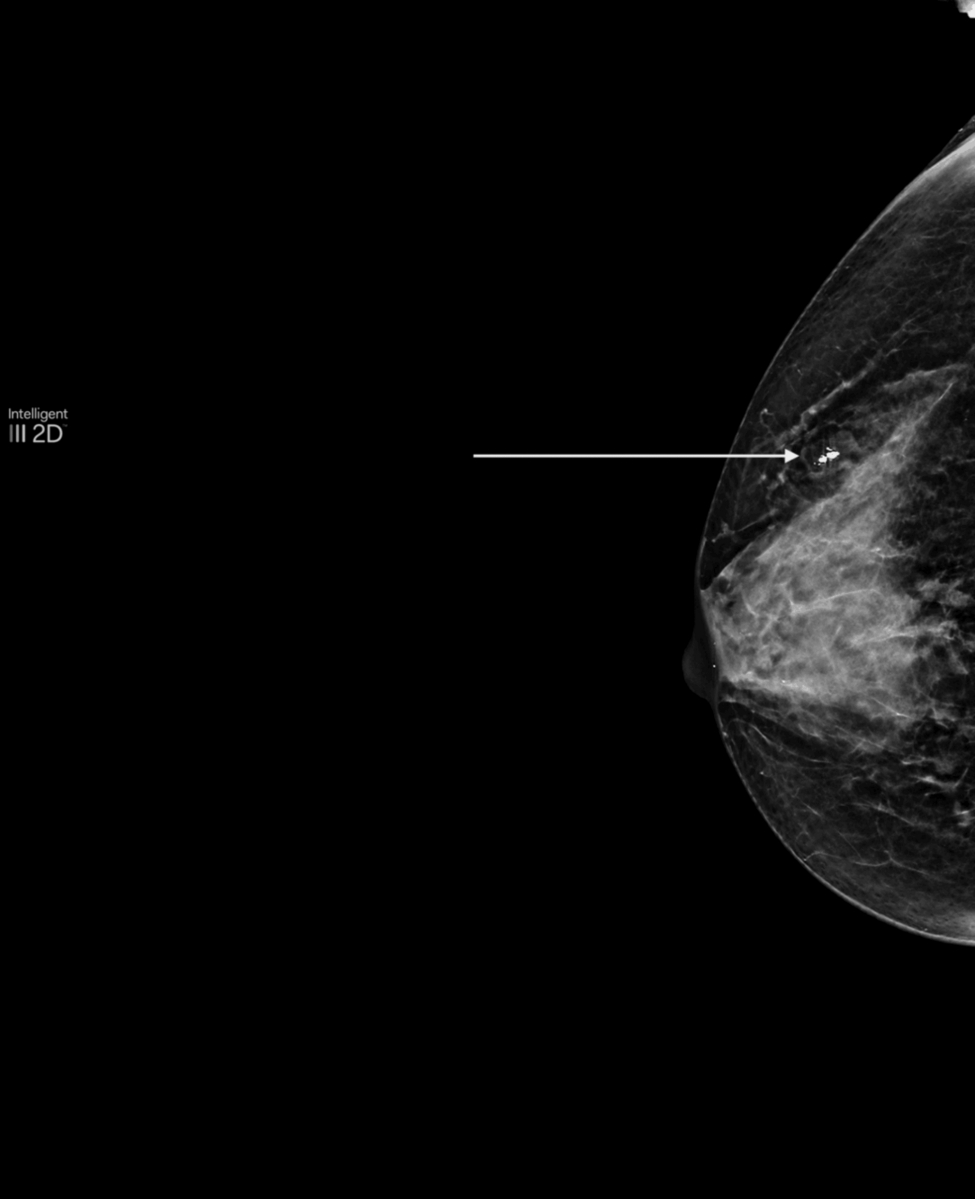
Mammogram revealing a fibroadenoma (arrow)

The left breast ultrasound demonstrated a huge mixed solid and cystic mass occupying the whole breast (Figure 3). It was difficult to measure as the mass extended beyond the footprint of the biconvex probe. The left axilla was also difficult to assess as it was obscured by the lesion. Both the ultrasound and mammogram on the right side only showed a small 1 cm calcified fibroadenoma with a clear right axilla (Figures 4-5). Mammogram and MRI assessment of the left breast was technically not feasible. The lesion was documented as breast imaging reporting and data system (BI-RADS) V, and we processed it with a PET-CT for further assessment.

**Figure 6:**
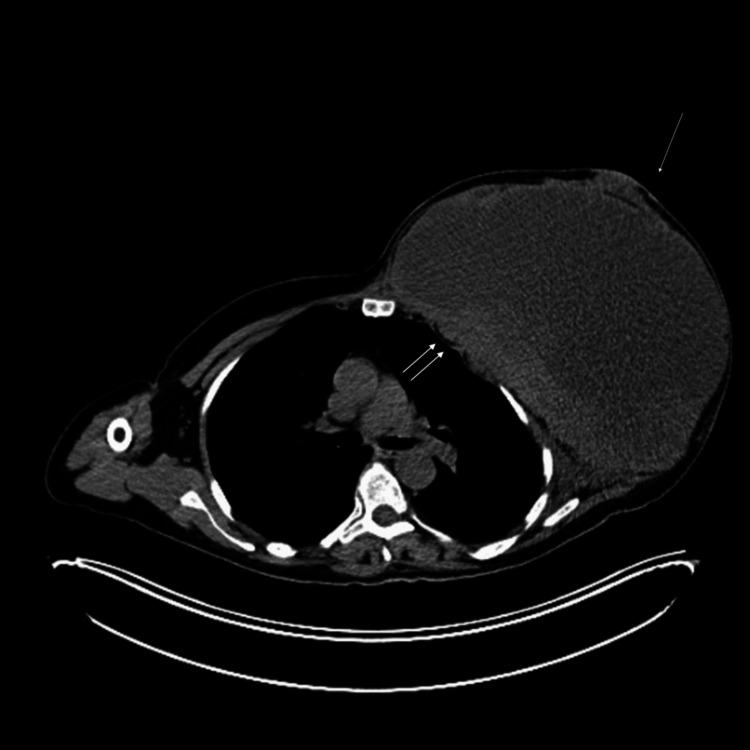
The PET-CT revealing a huge left breast mass. The single arrow points at the lesion, and the double arrows point at the attachment and invasion to the chest wall.

**Figure 7:**
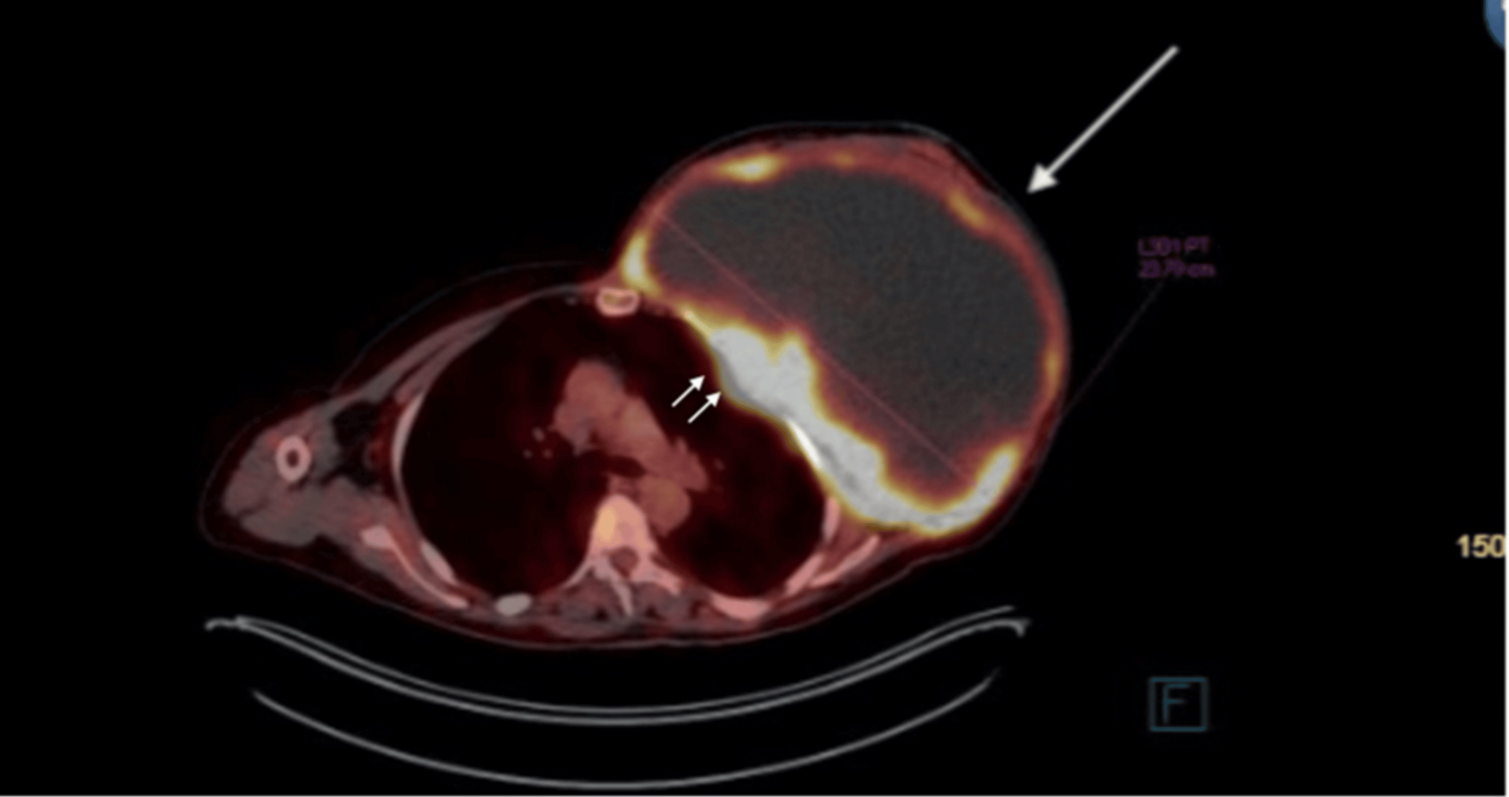
Huge left breast mass observed on the PET-CT with high peripheral FDG uptake The single arrow points at the lesion, and the double arrows point at the attachment and invasion to the chest wall. FDF: Fludeoxyglucose-18

The PET-CT illustrated a large, multilobulated, heterogeneous hypoattenuating mass with intense peripheral fludeoxyglucose-18 (FDG) uptake (Figures 6-7). There was infiltration in the chest wall and FDG-avid nodular pleural thickening. No separable regional or distant FDG-avid lymphadenopathy was identified. The lungs revealed an FDG-avid pulmonary nodule in the left perihilar region, likely thoracic in origin. No other suspicious lung or other organ metastases. However, there was diffuse moderate FDG uptake involving the sternum, ribs, spine, and proximal appendicular skeleton. While this could be reactive, involvement cannot be excluded.

Left breast core needle biopsy revealed high-grade sarcoma with smooth muscle differentiation. Multiple immunohistochemical stains were used; only desmin was positive, while myogenin, MyoD1, pan CK, EMA, CK5/6, p63, S100, and CD31 were all negative. According to the pathologist, the initial biopsy sample was insufficient; the excisional biopsy was needed for confirmation. According to the triple assessment findings, the differential diagnosis of the lesion was thought to be a sarcomatoid component of invasive ductal carcinoma (IDC), malignant phyllodes tumor, or primary leiomyosarcoma of the breast, respectively.

The case was discussed by the multidisciplinary team at our institution, and it was agreed that the lesion is unresectable at this stage. Therefore, the team decided to go with palliative systemic therapy and reassess response and resectability. The patient is currently on chemotherapy in the form of ifosfamide 2500 mg/m2 D1-D3, doxorubicin 25 mg/m2 D1-D3, and mesna.

## Discussion

The approach to any breast mass should include the triple assessment: clinical history and examination, radiological imaging, and pathological assessment as needed. For patients less than 30 years old, the recommended radiological modality is ultrasound. Mammography is recommended for patients aged more than 40 years old, while patients between 30 and 40 years of age may be evaluated first by ultrasound followed by a diagnostic mammogram. The images should then be used for staging per BI-RADS [9]. Another modality recommended to assess the breast is MRI [10]. However, in some cases, especially with giant breast lesions, a triple assessment may not help reach a final diagnosis, and the diagnosis is only revealed after surgery.

In our case, the mammogram and MRI were not technically feasible. The ultrasound, although it showed a subspinous lesion, was inconclusive. Hence, we proceeded with the PET-CT. It revealed malignant features, and the core needle biopsy failed to give a conclusive diagnosis. 

Giant breast tumors are any breast lesions that are more than 5 cm [5]. Although giant breast malignancies are uncommon, they are frequently reported in the literature with different causes [2,3,11]. While giant masses are any mass more than 5 cm, hugely giant masses are rarely reported; Islam et al. reported the case of the largest neglected giant phyllode tumor that reached 50 by 50 cm [12]. A diverse range of benign and malignant breast lesions can lead to single or numerous large lumps that include phyllodes tumors, lipomas, abscesses, carcinomas, cysts, fibroadenomas, hamartomas, or hematomas [13]. Even intraductal papilloma has been reported in a case report of a giant breast mass that was challenging to manage [14].

Breast sarcomatoid carcinoma is a differential diagnosis in our case. It arises from the mesenchymal tissue of the breast. It needs a high level of suspicion as it is a very rare and highly aggressive tumor; it has rapid growth, high recurrence, early hematogenous metastasis, and poor prognosis [15,16]. It occurs across varied age groups; Liuni et al. [15] found the age of presentation to be between 22 years and 91 years. Furthermore, due to its aggressiveness, it may require more than one modality of treatment [15]. By reviewing the literature, we found some challenging cases of breast sarcomas where the preoperative diagnosis failed to be reached [2,3]. Stromal sarcomas of the breast are estimated to be about 1% of all breast malignant tumors [17].

Another differential in our case is the malignant phyllodes tumor, which is a rare tumor of the connective tissue of the breast. Phyllodes tumors tend to rapidly grow to a huge size, but they seldom invade other parts of the body. Although the majority are benign, less than 1% of all breast cancers are found to be malignant phyllodes tumors. Surgery is the definitive treatment for phyllodes tumors; however, they may recur [11]. It is usually found in women aged between 40 and 50 [18]. Studies have shown that its preoperative diagnostic attempts have a low accuracy rate [4].

Breast leiomyosarcoma is an extremely rare tumor and is a differential in our case. The primary leiomyosarcoma of the breast accounts for not more than 1% of all malignancies of the breast and not more than 5% of the entire sarcoma of soft tissue. The patients' ages range from 24 to 86 years old. The tumor commonly ranges from 1 cm to 9 cm. According to Oktay et al., only 16 cases have been documented in the literature [17]. In 1968, Waterworth et al. published a case of fibrosarcoma; however, the tumor displayed characteristics of the traditional leiomyosarcoma. Therefore, it is believed to be the first reported case of leiomyosarcoma of the breast [17,19].

The diagnosis of giant tumors is not the only challenge; treatment appears to be another hurdle. As the lesions are aggressive, they may require a combative course of therapy that includes surgery, chemotherapy, and even radiotherapy to decrease local recurrence [20]. The challenge of local recurrence may interfere with the reconstruction of the breast. Saxena et al. reported a case of recurrent phyllodes tumor in the reconstructed flap [21]. In our case, surgery is required to reveal the diagnosis. However, the lesion is unresectable. Therefore, the patient is currently undergoing chemoradiotherapy to reduce the size and local aggressiveness of the lesion.

## Conclusions

Giant breast malignancies are uncommon. They include numerous differentials, and their management is usually challenging, as reaching a diagnosis is not easy without sufficient tissue for pathological diagnosis. We believe new diagnostic guidelines need to be adopted for such cases, as the classical approach can fail at arriving at the diagnosis. The adjusted guidelines for such lesions may incorporate early involvement of a multidisciplinary team and incorporation of PET-CT or MRI if feasible. For the biopsy, multiple core needle vacuum-assisted biopsies for deeper tissue may be required, and excisional biopsy should be planned for earlier. Planning for neoadjuvant therapy might aid in downstaging earlier.
